# Chemical composition and antibacterial activity of the essential oils of *Ferula vesceritensis* Coss et Dur. leaves, endemic in Algeria

**DOI:** 10.1186/2191-2858-2-31

**Published:** 2012-09-03

**Authors:** Amar Zellagui, Noueddine Gherraf, Salah Rhouati

**Affiliations:** 1 Laboratory of Biomolecules and Plant Breeding, Life Science and Nature Department, Faculty of Exact Science and Life Science and Nature, University of Larbi Ben Mhidi, Oum El Bouaghi, Algeria; 2 Laboratory of Natural Products and Organic Synthesis, Department of Chemistry, Faculty of Science, University of Mentouri-Constantine, Constantine, Algeria

**Keywords:** *Ferula vesceritensis*, Volatile oils, GC-MS, Antimicrobial activity

## Abstract

****Background**:**

The biological importance of members of genus *Ferula* promoted us to investigate the leaves of *Ferula vesceritensis* Coss et Dur. (endemic plant) previously not investigated. This study presents the chemical composition and antibacterial activities of the hydrodistilled oils.

****Results**:**

Volatile components of the leaves of *F. vesceritensis* have been studied by gas chromatography–mass spectrometry to afford 23 compounds. The major components were found to be 5,9-tetradecadiyne (24.72%), germacrene D (24.51%), farnesene (8.57%), and α-bisabolene (8.57%). The antimicrobial activities of the essential oils were evaluated by disk diffusion method and tested against Gram-positive and Gram-negative bacteria. The volatile oil showed a strong antibacterial activity against *Staphylococcus aureus, Escherichia coli*, and *Klebsiella pneumonia.*

****Conclusions**:**

These results reinforce the previous studies showing that the genus *Ferula* is considered as a good source of essential oils. The results presented here can be considered as the first information on the antimicrobial properties of *F. vesceritensis.*

## **Background**

Since the middle ages, essential oils have widely been used for bactericidal, virucidal fungicidal, antiparasitical, insecticidal, medicinal, and cosmetic applications, especially nowadays in pharmaceutical, sanitary, cosmetic, and agricultural and food industries. Because of the mode of extraction, mostly by hydrodistillation from aromatic plants, they contain a variety of volatile molecules such as terpenes and terpenoids, phenol-derived aromatic components, and aliphatic components [1].

The exclusively old-world genus *Ferula*, belonging to the family *Apiaceae*, has some 130 species distributed throughout the Mediterranean area and Central Asia. These plants are often used as spices and in the preparation of local drugs. The resins are reported to be used for stomach disorders such as a febrifuge and carminative agent [2]. Some species are used in traditional medicine for the treatment of skin infections [3] and hysteria [2]. Previous study dealing with members of this genus revealed that the main constituents are sesquiterpenes and sesquiterpene coumarins. More than 70 species have been studied chemically leading to the fact that germacranes, humulanes, carotanes, himachalanes, and guaianes represent the main sesquiterpene constituents of the genus [4-10]. *Ferula* spp. are also known for their toxicity and pharmacology. Daucane esters from *F. communis* and *Ferula arrigonii* showed antiproliferative activity on human colon cancer lines [11] and calcium ionophoretic and apoptotic effects in the human jurkat T-cell line [12].

*Ferula vesceritensis* belongs to umbelifereae family, which widely spread in north Africa, this plant is abundant in south east of Algeria. The genus *Ferula* represented in Algeria by six species [13].

*Ferula vesceritensis* is indigenous to Algerian Sahara. According to ethnobotanical investigation, fruit decoction is used in folk medicine to treat headaches, fever, and throat infections, while the livestock avoids grazing it [14].

Our continuation of investigation carried out on *F. vesceritensis*[15-17]. The essential oils of the leaves of *F. vesceritensis* led to the identification of 23 compounds. Moreover, the evaluation of the antibacterial activity of the essential oils revealed a very important effect against some bacteria strains.

## **Methods**

### **Gas chromatography/mass spectroscopy**

GC/MS analysis was carried out on a Thermoquest-Finnigan Trace GC/MS instrument equipped with a DB-1 fused silica column (30 m, 0.25 mm i.d., film thickness 0.25 m). The oven temperature was raised from 60 to 250°C at a rate of 5°C/min then held at 250°C for 10 min; transfer line temperature was adjusted at 250°C. Helium was used as the carrier gas at a flow rate of 1.1 mL/min with a split ratio of 1/50. Identification of the constituents of each oil was achieved by comparison of their mass spectra and retention times (Rt) with those reported in the literature, and those of authentic samples.

### **Antimicrobial activity**

The antibacterial activity test was carried out on essential oils of the leaves of *F. vesceritensis* roots using disk diffusion method (NCCLS) against four human pathogenic bacteria, including Gram positive, Gram-negative bacteria.

## **Results and discussion**

This study focused essentially on the phytochemical and antibacterial screening of *F. vesceritensis*. The specie has been screened for seven chemical groups. The analyses reveal the presence of volatile oils, flavonoids, saponins, tannins, carotenoids, and coumarins (Table 1).

**Table 1:** **Phytochemical screening from *F. vesceritensis***

**Chemical groups**	**Roots**	**Leaves**	**Stems**	**Flowers**	**Fruits & seeds**
Volatile oils	++	+++	++	+++	+++
Carotenoids	+	+	+	+	++
Alkaloids	–	–	–	–	–
Flavone aglycones	+	+	+−	+	+−
Coumarins	+++	+++	+++	+++	+++
Tanins	+	+	+	+	+
Saponins	++	–	–	–	–
Flavone glycosides	–	++	++	++	++

Essential oils from the leaves of *F. vesceritensis* have been studied by GC–MS to afford 23 components. The yield was 1.82% on dry weight basis. In previous studies, the essential oil obtained from the roots of *Ferula ferulaoides* yielded 2.4–3.2% of essential oil from dry roots [18] and average 1,66–3.85% in the fruits of *Ferula gummosa*[19]. The essential oil components identified from *F. vesceritensis* are listed in Table 2; the major components were found to be 5,9-tetradecadiyne 24.72%, germacrene D (24.51%), farnesene (8.57%), α-bisabolene (8.57%). Some other compounds were only present in minor amounts.

**Table 2:** **Chemical composition of essential oils from *F. vesceritensis***

**Compounds**	**Rt**	**%**
Ocimene	07.105	0.31
Limonene	10.554	0.12
Fuseloel	13.587	0.11
Nerylacetone	17.852	**4.45**
Dihydrocarvyl acetate	20.183	**6.20**
Z-ocimene	22.290	03.19
α-methyl pentenal	23.193	0.42
5,9-tetradecadiyne	24.851	**24.72**
1,1-methylene-3-(propenylidene)-5-vinylcyclohexane	25.026	0.69
Calarene	25.173	2.59
Farnesene	25.358	2.71
α-bisabolol	26.632	0.89
Dihydrocarveol acetate	26.769	1.59
A-Bisabolene	26.925	0.53
Nerolidol Rep	27.203	1.02
Xanth ou α farnesene	27.402	2.00
citral	27.595	0.89
Cububene	27.902	**8.57**
Germacrene D	28.311	**24.51**
Nerolidol	31.634	1.55
Bisabolol	32.213	**8.57**
linalol	34.691	4.35
Total		**99.98**

Bold entries highlight the major components.

Concerning the chemical composition of the essential oil of other *Ferula* species, Shatar [18] showed that the roots of *F. ferulaoides* growing in Mongolia were dominated by Guaiol (58.76%), and (*E*)-nerolidol (10.16%). In the fruits of *F. gummosa* from Iran, the major components were β-pinene (43.78%), α-pinene (27.27%) [20], also found β-pinene (43.78%), α-pinene (27.27%) of *F. gummosa* growing in Isfahan [19], also reported that the essential oil of the *Ferula latisecta* collected in Iran was characterized by high contents of (Z)-Ocimenone (32.4%), (E)-ocimenone (20.3%), and *cis*-pinocarvone (11.4%) [21].

### **Antibacterial activity**

The antimicrobial activities and toxicity of essential oil have been documented, but their modes of action are complex and still in some cases unknown, considering the large number of different groups of chemical compounds present, this activity is due to the presence of active constituents, mainly attributable to isoprenes such as monoterpenes, sesquiterpenes, and related alcohols, other hydrocarbons and phenols [1].

The diffusion test was applied to four microorganisms including Gram-positive, -negative bacteria. The results summarized in Table 3 showed that the volatile oil from *F. vesceritensis* prevented the growth of all the tested microorganisms and it has been revealed that the medium diameter of inhibition zone increase proportionally with the increase of concentrations.

**Table 3:** **Inhibition effect of essential oils from *F. vesceritensis***

**Bacteria**	**250 μg/mL**	**500 μg/mL**	**1000 μg/mL**	**2000 μg/mL**	**4000 μg/mL**	**8000 μg/mL**
***Escherichia coli ATCC 25922***	14.5 ± 0.75	19.0 ± 0.95	19.0 ± 0.81	21.5 ± 0.57	22.0 ± 0.57	26 ± 1.25
***Klebsiella pneumonia***	11.5 ± 0.0	13.5 ± 0.5	15.0 ± 0.95	20.5 ± 0.95	22.5 ± 0.95	24.5 ± 0.57
***S. aureous***	12.5 ± 0.57	14 ± 0.57	14.5 ± 0.5	17 ± 1.21	19.5 ± 1.29	27 ± 0.81
***Pseudomonasaerugenosa ATCC 27853***		06.00	7 ± 1.73	11 ± 0.81	13.5 ± 1.15	16 ± 1.41

The obtained inhibition zone varied from 6.00 to 27.00 mm with a highest inhibition zone recorded with *Staphylococcus aureus* at 8 mg mL and with 26 mm at *E. coli* in the same concentration. This results corresponding with those obtained on *F. gummosa* and *F. latisecta*[20]. It should be mentioned that there are no background antibacterial studies on *F. vesceritensis.*

## **Experimental**

### **Plant material**

The leaves of *F. vesceritensis* were collected on May 2010 near Ghardaya Algeria. The plants were identified by Dr. M. Chahma, Faculty of Sciences, University of Ourgla, Algeria, voucher specimens were deposited at the Chemistry Department, University of Mentouri-Constantine under code number (AM#112).

### **Extraction**

Essential oils were obtained by hydrodistillation of 150 g of dried aerial parts using a Clevenger-type apparatus for 3 h. Diethyl ether (10 mL) was used as the collector solvent as reported in literature. After evaporation of the solvent, the oil was dried over anhydrous sodium sulfate and stored in sealed vials protected from the light at −20°C before analyses. Three oil samples were obtained by hydrodistillation and subsequently analyzed by GC-MS.

## **Antimicrobial activity**

### **Microorganism strains**

All of the bacteria; standard strains *E. coli* ATCC 25922, *P. aerugenosa* ATCC 27853 and (clinical stains: *S. aureus, K. pneumonia*) were obtained from Bacteriology Laboratory Constantine Hospital University (C.H.U).

The bacterial strains were first grown on Muller Hinton medium (MHI) at 37°C for 24 h prior to seeding on to the nutrient agar. A sterile 6-mm-diameter filter disk (Whatman paper no. 3) was placed on the infusion agar seeded with bacteria, and each extract suspended in water was dropped on to each paper disk (40 μL per disk) for all of prepared concentrations (8, 4, 2, 1, 0.5, 0.25 mg/mL). The treated Petri dishes were kept at 4°C for 1 h, and incubated at 37°C for 24 h. The antibacterial activity was assessed by measuring the zone of growth inhibition surrounding the disks. Each experiment was carried out in triplicate.

## **Conclusions**

Our study of the Algerian *F. vesceritensis* leaves led to the extraction and characterization of 23 compounds followed by the evaluation of antimicrobial activity for the first time. These results reinforce the previous studies showing that the genus *Ferula* is considered as a good source of essential oils. The results presented here can be considered as the first information on the antimicrobial properties of *F. vesceritensis.*

## **Competing interests**

The authors declare that they have no competing interests.
